# Computational Analysis of Thermal Adaptation in Extremophilic Chitinases: The Achilles’ Heel in Protein Structure and Industrial Utilization

**DOI:** 10.3390/molecules26030707

**Published:** 2021-01-29

**Authors:** Dale L. Ang, Mubasher Zahir Hoque, Md. Abir Hossain, Gea Guerriero, Roberto Berni, Jean-Francois Hausman, Saleem A Bokhari, Wallace J. Bridge, Khawar Sohail Siddiqui

**Affiliations:** 1 Department of Molecular Sciences, Macquarie University, North Ryde, NSW 2109, Australia; dale.ang@mq.edu.au; 2 Bio-Bio-1 Research Foundation, Sangskriti Bikash Kendra Bhaban, 1/E/1 Poribagh, Dhaka 1000, Bangladesh; mubasher_zahir856@hotmail.com (M.Z.H.); mdabir14@gmail.com (M.A.H.); 3 Department of Biochemistry and Microbiology, North South University, Plot 15, Block B, Bashundhara, Dhaka 1229, Bangladesh; 4 Research and Innovation Department, Luxembourg Institute of Science and Technology, 5, rue Bommel, Z.A.E. Robert Steichen, L-4940 Hautcharage, Luxembourg; jean-francois.hausman@list.lu; 5 TERRA Teaching and Research Center, Gembloux Agro-Bio Tech, University of Liège, 5030 Gembloux, Belgium; Roberto.Berni@uliege.be; 6 Biosciences Department, COMSATS University Islamabad, Park Road, Islamabad 45550, Pakistan; saleem.a.bokhari@comsats.edu.pk; 7 School of Biotechnology and Biomolecular Sciences (BABS), University of New South Wales, Sydney, NSW 2052, Australia; wj.bridge@unsw.edu.au

**Keywords:** chitinases, thermostability, bioinformatics, cold-adapted, thermophilic, biotechnology, enzyme, protein structure–function–stability, molecular dynamics (MD) simulations, industrial applications

## Abstract

Understanding protein stability is critical for the application of enzymes in biotechnological processes. The structural basis for the stability of thermally adapted chitinases has not yet been examined. In this study, the amino acid sequences and X-ray structures of psychrophilic, mesophilic, and hyperthermophilic chitinases were analyzed using computational and molecular dynamics (MD) simulation methods. From the findings, the key features associated with higher stability in mesophilic and thermophilic chitinases were fewer and/or shorter loops, oligomerization, and less flexible surface regions. No consistent trends were observed between stability and amino acid composition, structural features, or electrostatic interactions. Instead, unique elements affecting stability were identified in different chitinases. Notably, hyperthermostable chitinase had a much shorter surface loop compared to psychrophilic and mesophilic homologs, implying that the extended floppy surface region in cold-adapted and mesophilic chitinases may have acted as a “weak link” from where unfolding was initiated. MD simulations confirmed that the prevalence and flexibility of the loops adjacent to the active site were greater in low-temperature-adapted chitinases and may have led to the occlusion of the active site at higher temperatures compared to their thermostable homologs. Following this, loop “hot spots” for stabilizing and destabilizing mutations were also identified. This information is not only useful for the elucidation of the structure–stability relationship, but will be crucial for designing and engineering chitinases to have enhanced thermoactivity and to withstand harsh industrial processing conditions

## 1. Introduction

Endochitinases (EC 3.2.1.14) randomly hydrolyze internal glycosidic linkages of insoluble chitin, which is a linear polymer of β-1,4-linked *N*-acetylglucosamine residues of soluble low molecular weight chitooligosaccharides (COSs) that are found in viruses, bacteria, archaea, plants, and animals, including mammals. In humans, chitinases were suggested to be involved in the defense against fungi and other pathogens, as well as in inflammation [1]. After cellulose and lignin, chitin is the most abundant polymeric waste on Earth and is derived from the exoskeletons of marine invertebrates, crustaceans, insects, and fungi. Compared to cellulases, chitinases have been biotechnologically underexploited, but potential applications that are currently being explored [2,3,4] include fungicides and insecticides for use in agriculture, the bioconversion and bioremediation of chitin waste into single-cell protein, and value-added COSs and human health care treatments.

Much work has been reported on the catalytic mechanism of chitinases [4,5,6]; however, few reports have explored the mechanism of stability. To date, a handful of thermally adapted chitinases from extremophilic organisms have been described [7,8,9], and currently, the X-ray structures of only two cold-adapted (*Moritella marina* [10,11]; *Arthrobacter* sp.) and a single thermophilic (*Pyrococcus furiosus* [12]) chitinases have been reported. The X-ray structure of the P-As from Antarctic *Arthrobacter* sp. has yet to be reported. Surprisingly, the X-ray structures of all thermally adapted chitinases have been discussed from a functional rather than a stability perspective.

Numerous features have been correlated with thermal adaptation in proteins; however, every cold-adapted or thermophilic protein is associated with a unique set of compositional and structural features that govern its stability [13,14,15]. Identifying those unique structural features that influence the stability in a thermally adapted set of homologous proteins has been a topical area of research with the view to defy activity–stability trade-off [13,14] in enzymes. The use of rational design [16] or chemical modification [13,17,18] has been employed to elucidate the structure–function–stability relationships in enzymes and to enhance their stability to suit various biotechnological applications [19]. Understanding the role of the structural features implicated in the stability of enzymes is crucial for designing thermostable chitinases for various biotechnological applications.

In this study, chitinases belonging to the GH18 family were chosen because of their wide distribution in nature, where they perform a diverse range of functions. Due to the importance of GH18 chitinases in the efficient degradation of chitin, extensive work has been performed, including using high-resolution X-ray imaging to identify the structures of thermally adapted homologs [20,21]. Although many thermostable GH18 chitinases have been described for industrial applications, the molecular basis of thermostability is lacking [20]. To the best of our knowledge, our study is the first to provide in silico proofs of the biochemical features conferring high stability to hyperthermophilic chitinases from the GH18 class compared to mesophilic and psychrophilic counterparts and to show that the extended floppy loops on the surface of cold-adapted and mesophilic enzymes act as “weak spots” by initiating unfolding.

Detailed bioinformatics analysis of five thermally adapted chitinases from the GH18 family was carried out in order to identify compositional and structural features that influence stability. Comprehensive molecular dynamics (MD) simulations and analysis were performed with the aim of identifying the basis for the thermal behavior, including any regions of instability or flexibility from where unfolding might be initiated. From this, loop regions were identified using principal component analysis (PCA) and root-mean-square fluctuations (RMSF) analysis, followed by visual inspection to determine the significance for chitinase stability and its relationship to the active site. Critical residues that impart stability or instability were identified and mutations that may stabilize chitinases were suggested for future protein engineering approaches.

## 2. Results

**Figure 1:**
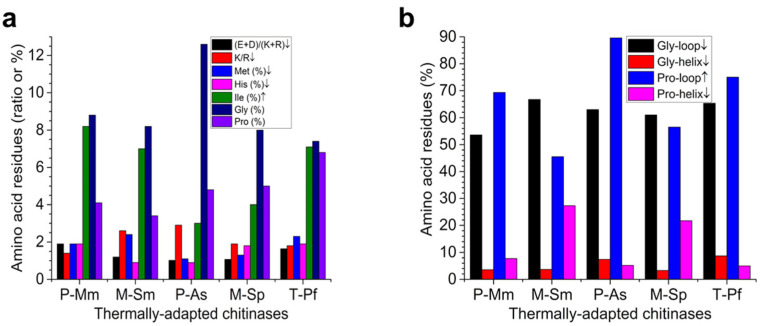
Compositional features in thermally adapted chitinases. (**a**) The relative number of amino acid residues (ratio or %). (**b**) Gly and Pro in the secondary structures (%). All features were analyzed using the ProtParam tool or PDB DeepView. ↑, higher value of the parameter is associated with higher stability; ↓, higher value of the parameter is associated with lower stability. P, psychrophilic; M, mesophilic; T, thermophilic. Mm, *M. marina*; Sm, *S. marcescens;* As, *Arthrobacter* sp.; Sp, *S. proteamaculans*; Pf, *P. furiosus.*

**Figure 2:**
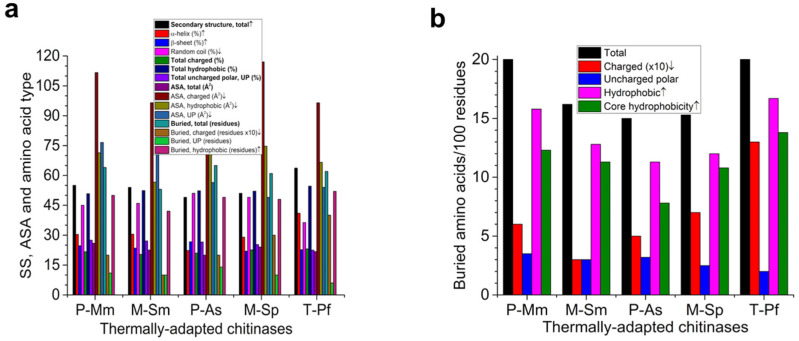
Compositional and structural features in thermally adapted chitinases. (**a**) Secondary structure (SS) elements, accessible solvent area (ASA), types of amino acids, and total buried residues. (**b**) Various types of buried amino acids normalized to per 100 residues. SS elements were found using X-ray structures; total charged (DEHKR), hydrophobic (GAVLIPMFW), and uncharged polar (CNQSTY) components were found using ProtParam; average ASA and various types of buried residues were found using WHATIF. ↑, higher value of the parameter is associated with higher stability; ↓, higher value of the parameter is associated with lower stability. P, psychrophilic; M, mesophilic; T, thermophilic. Mm, *M. marina*; Sm, *S. marcescens;* As, *Arthrobacter* sp.; Sp, *S. proteamaculans*; Pf, *P. furiosus.*

**Figure 3:**
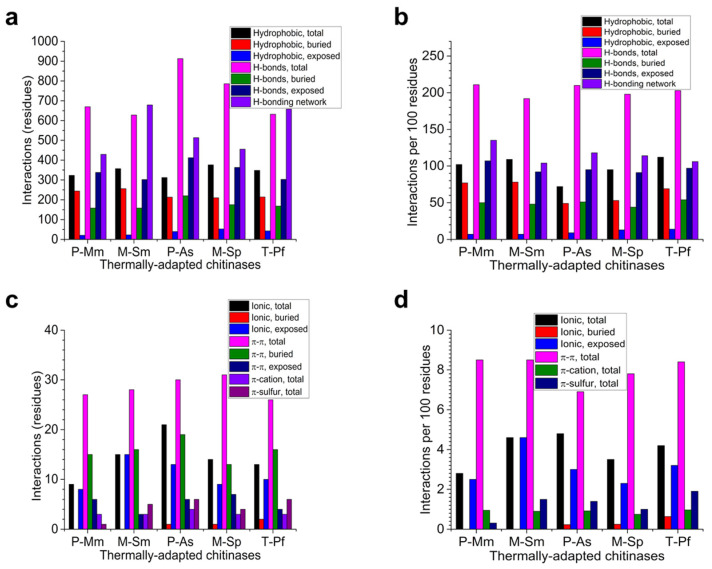
Various interactions in thermally-adapted chitinases. (**a**) Total hydrophobic interactions and hydrogen bonds (H-bonds). (**b**) Normalized hydrophobic interactions and hydrogen bonds per 100 residues. (**c**) Total ionic and pi interactions. (**d**) Normalized ionic and pi interactions per 100 residues. Hydrophobic interactions (within 5 Å), H-bonds (dA:O, N, 3.5; S, 4.0 Å), ionic/salt bridges (within 4 Å), π-π (4.5–7.0 Å) and π-sulfur interactions (within 5.3 Å) were found using PIC; π-cation interactions (within 6 Å) were found using realistic electrostatics (CaPTURE) and the optimal H-bonding network was found using WHATIF. Higher values of interactions are associated with higher stability. P, psychrophilic; M, mesophilic; T, thermophilic. Mm, *M. marina*; Sm, *S. marcescens;* As, *Arthrobacter* sp.; Sp, *S. proteamaculans*; Pf, *P. furiosus.*

The multiple alignments of thermally adapted chitinases showed that cold-adapted P-Mm (from *M. marina*) and P-As (from *Arthrobacter* sp.) have 56 and 35% identity with their mesophilic homologs, M-Sm (from *Serratia marcescens)* and M-Sp (from *Serratia proteamaculans*), respectively. However, T-Pf (from *P. furiosus*) showed only ≈17–20% identity with all other chitinases. The bioinformatics analysis findings are presented in Figure 1, Figure 2 and Figure 3 and Table 1 and Table S1.

### 2.1. Correlation between Compositional Features and Stability in Chitinases

The compositional features of all five chitinases are given in Figure 1. Their thermal stabilities (T_m_, T_opt_, and thermal inactivation parameters), which are consistent with their psychrophilic, mesophilic, and hyperthermophilic classifications, along with other compositional/structural features, are given in Table 1. The correlation of the compositional features with thermal stabilities are represented by arrows, where an up arrow (↑) indicates that the higher value is associated with a higher stability and a down arrow (↓) indicates that the higher value of the element is associated with lower stability. Based on X-ray structure analysis, both psychrophilic and M-Sp were monomeric, whereas M-Sm and T-Pf were dimeric (Table 1).

The lower pI was predominantly due to a higher (D + E)/(K + R) ratio and was correlated with the higher flexibility and a concomitant lower stability. Interestingly, cold-adapted P-Mm and P-As were the most and least negatively charged proteins, respectively. However, compared to its mesophilic homolog (M-Sm), cold-adapted P-Mm was significantly more negatively charged, whereas no difference was found between cold-adapted P-As and mesophilic M-Sp. However, contrary to the rule described above, hyperthermophilic T-Pf showed a pI that was lower than all chitinases, except P-Mm (Figure 1a, Table 1).

Due to the ability of Arg residues to form multiple electrostatic interactions, a higher R/K ratio was correlated with higher stability. Cold-adapted P-As showed a lower R/K (i.e., highest K/R ratio) ratio relative to its mesophilic homolog (M-Sp), which was consistent with the thermolabile nature of P-As. In contrast, the cold-adapted P-Mm showed a higher R/K ratio relative to its mesophilic homolog (M-Sm). The value for hyperthermophilic T-Pf was closer to that of the mesophilic M-Sp, implying that the extreme stability of T-Pf was not due to the higher number of Arg residues. Thermophilic T-Pf had fewer proline residues in its α-helices, which was consistent with its higher stability (Figure 1). A less negatively charged N-cap in cold-adapted P-As was consistent with its lower stability (Table 1). Except for the few compositional features identified above, no clear-cut trend was found between the compositional features and the stability of chitinases (Figure 1, Table 1).

### 2.2. Correlation between Structural Features and Stability in Chitinases

Figure 2 and Table 1 show the structural features of all five thermally adapted chitinases. The thermophilic chitinase (T-Pf) showed the highest percentage of total secondary structure elements, whereas the cold-adapted P-As the lowest, which is consistent with their stability. The thermostable T-Pf had the highest content of α-helices and the cold-adapted P-As had the lowest. Conversely, the random coil content was the lowest in T-Pf and the highest in P-As. No association of stability with the β-sheet content was observed (Figure 2a).

The total and specific contents (per 100 residues) of hydrophobic-buried residues and core hydrophobicity also showed the highest and lowest values for T-Pf and P-As, respectively, which was consistent with their thermophilic and psychrophilic stability profiles (Figure 2). Interestingly, T-Pf and P-As showed 3 and 6 Gly residues buried inside the core (Table 1).

The volume of cavities increased in the order P-Mm < T-Pf < M-Sm < M-Sp < P-As, suggesting no clear-cut trend. Though both the cold-adapted P-As (4206 Å^3^) and the mesophilic M-Sp (4135 Å^3^) showed the highest cavity volumes, the cavity volume of cold-adapted P-Mm was lower (1001 Å^3^) in comparison to the volume (1495 Å^3^) of the dimeric thermophilic T-Pf (Table 1). Other structural parameters, such as the accessible solvent area, showed no clear association with stability (Figure 2a; see Section 2.4 for more details).

### 2.3. Correlation between Electrostatic, Hydrophobic, and Pi Interactions and Stability in Chitinases

Higher numbers of electrostatic interactions are generally correlated with a higher protein stability [13,20,21]. Figure 3 gives the overall analysis of the electrostatic, hydrophobic, and pi interactions and Table S1 gives more details about these interactions. Only cold-adapted chitinases have a single disulfide bridge (P-Mm, exposed; P-As, buried). Although the hydrophobic interactions per 100 residues were the highest for the thermophilic T-Pf (112) and the lowest for the cold-adapted P-As (72), the proportion of buried hydrophobic interactions were the highest for the cold-adapted P-Mm (77) and the mesophilic M-Sm (78). In contrast, the exposed hydrophobic interactions were the highest in the thermophilic T-Pf (14) and mesophilic M-Sp (13).

The number of hydrogen bonds per 100 residues was the highest for both cold-adapted chitinases and the lowest for both mesophilic homologs. Importantly, the thermophilic T-Pf had more buried H-bonds, whereas the mesophilic M-Sp had the lowest number. In contrast, cold-adapted P-Mm had the highest number of exposed H-bonds and the mesophilic homologs had the lowest number (Figure 3a,b). Consistent with the above results, both cold-adapted chitinases had the optimal hydrogen bonding network per 100 residues, whereas T-Pf (106) was slightly better than M-Sm (Figure 3a,b).

Regarding salt-bridges per 100 residues, although the cold-adapted P-Mm formed the lowest number of interactions (2.8), the other cold-adapted chitinase P-As forms the highest number of these interactions (4.8). The thermophilic T-Pf formed 4.2 ionic interactions per 100 residues (Figure 3c,d). The distribution of salt-bridges between COO^−^ and Lys, Arg, and His are given in Table S1. While P-As formed a majority of its salt-bridges (12) with Lys residues, salt-bridges with Arg were the highest (8) in mesophilic homologs, with 7 each in P-As and T-Pf. It is noteworthy that the thermophilic homolog had the highest number of buried salt bridges and was the only homolog with 2 buried COO^−^-to-Arg interactions.

There was no clear-cut trend in the π–π, cation–π, and π–sulfur interactions (Figure 3c,d). It is interesting to note that inter-subunit interactions in the case of dimeric M-Sm and T-Pf showed trends that were contrary to general expectations (Table S1). Compared to the thermophilic T-Pf, the mesophilic M-Sm had a higher interface area, more interface residues, and more interactions. Compared to the cold-adapted P-Mm, the higher stability of the mesophilic homolog M-Sm could be explained in part due to its dimeric nature with 88 concomitant inter-subunit interactions (Table S1).

### 2.4. MD Analysis and Identification of Flexible Surface Loops

MD simulations were performed on all five structures at two temperatures: 300 and 400 K. The secondary structure (SS) content, backbone root-mean-square deviation (RMSD), solvent-accessible surface area (SASA), and radius of gyration (Rg) values are presented in Figures S1 and S2. At 300 K, the structures were observed to be stable with respect to these parameters. The RMSD analysis did reveal a sudden rise for P-As at ≈40 ns before stabilizing (Figure S2). P-Mm and M-Sm also exhibited sudden spikes in RMSD of ≈0.2 nm at around 200 ns. These fluctuations also corresponded with fluctuations in the SASA and Rg values. Overall, the structures fell into three distinct Rg values: P-As and M-Sp at ≈2.2 nm, P-Mm and M-Sm at ≈1.9 nm, and T-Pf at ≈1.8 nm. At 400 K all structural parameters steadily increased with particularly erratic RMSD behavior of M-Sm between 30 and 150 ns before stabilizing. The SASA and Rg values also exhibited large fluctuations. The time scales involved here were not long enough to reveal any specific unfolding events, but did highlight the relative flexibility of each structure, with T-Pf being the clear outlier.

**Figure 4:**
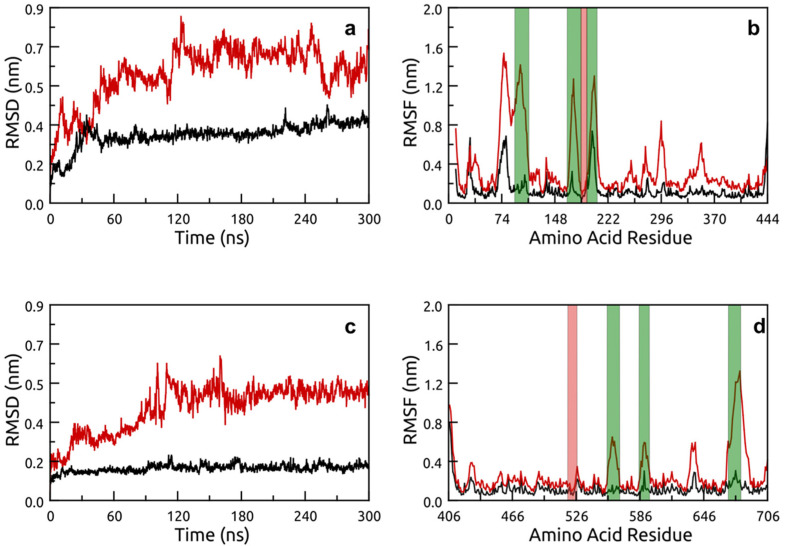
Backbone root-mean-square deviation (RMSD) and root-mean-square fluctuations (RMSF). (**a**,**b**) P-As and (**c**,**d**) T-Pf at 300 K (black) and 400 K (red). Identified primary floppy loops are highlighted in green with active sites in red.

The increased stability of thermophilic T-Pf at high temperatures was more evident when comparing the RMSD and RMSF plots with, for example, psychrophilic P-As. These data are presented in Figure 4 for both the backbone RMSD and residue RMSF values. At 300 K, the RMSD of T-Pf was below 0.2 nm for the entirety of the trajectory, with similar low RMSF values for all residues. At the higher temperature, the RMSD increased to under 0.5 nm and the RMSF values only increased for select regions that were distant from the active site (highlighted in red). In contrast, the P-As RMSD values were ≈0.4 nm, reaching closer to 0.9 nm at 400 K, with concomitant high RMSF values in regions flanking the active site. The RMSF values for all structures are presented in Figure S3 with the active site highlighted in red.

**Figure 5:**
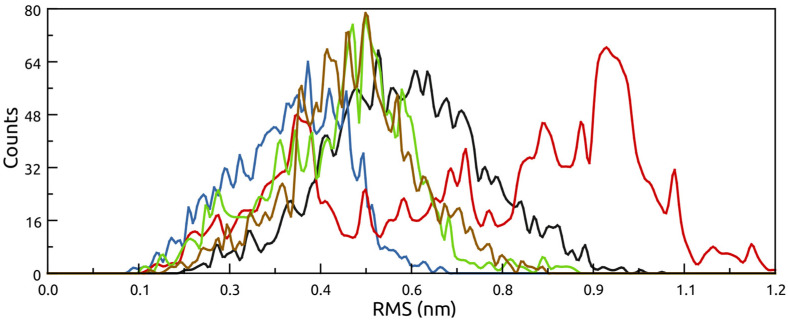
RMS matrix histogram showing the abundance of the conformer ensembles at 400 K. P-As (black), T-Pf (blue), M-Sm (red), P-Mm (green), M-Sp (brown).

Finally, clustering analysis was performed on the trajectories to determine the frequency of particular conformations as a function of the RMSD. These results are presented as a histogram of the RMS matrix descriptor (Figure 5). Here, all structures explored an RMS range that was concomitant with their thermal adaptation. For example, T-Pf was tightly clustered around 0.4 nm, while P-As covered a larger range from 0.1–0.9 nm. The one exception was M-Sm (Figure 5, red), which explored two clearly disparate conformations indicated by a peak at ≈0.4 nm and another larger peak at ≈0.9 nm.

**Figure 6:**
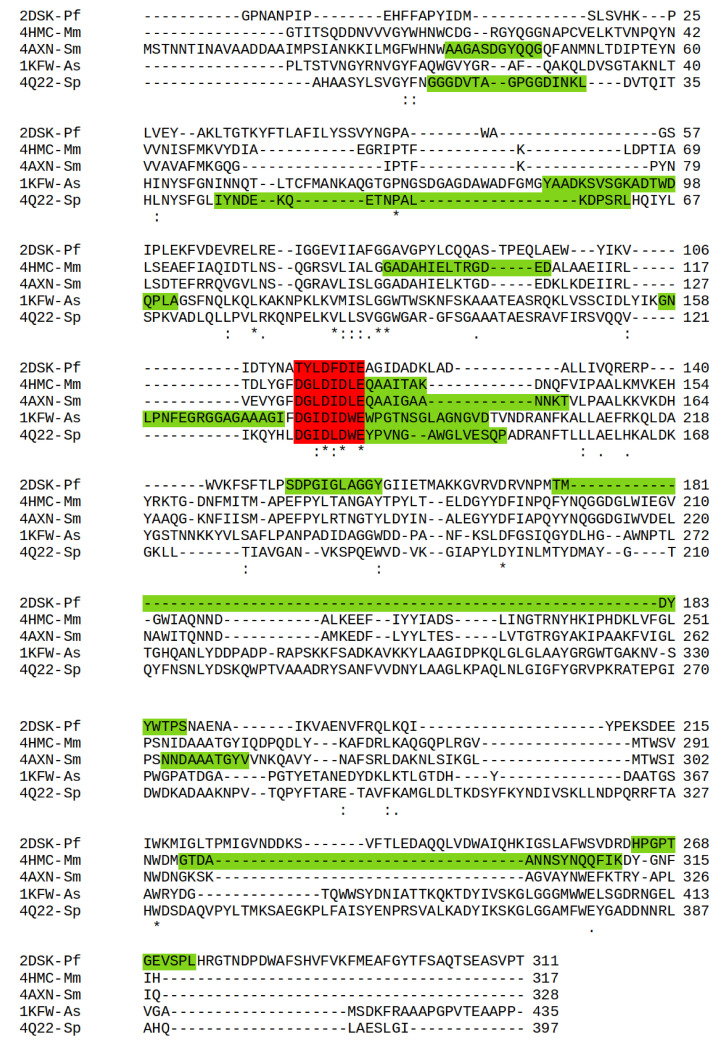
Multiple alignments of thermally adapted chitinases. Active-site residues (stars) within active sites (red) and loops (green). (*), Fully conserved residue; (:), conservation between groups of strongly similar properties; (.), conservation between groups of weakly similar properties.

T-Pf had the least number of identifiable loops (22) compared with 24 each in P-Mm and M-Sm, 26 in M-Sp, and 30 in P-As. Importantly, T-Pf possesses only three loops with more than 10 residues, whereas for the cold-adapted chitinases, P-As had eight and P-Mm had five such large loops. Mesophilic M-Sm and M-Sp both possessed such loops. Analysis of the MD trajectories identified the loops with the largest motion and their proximity to the active site. Figure 6 shows the primary identified loops and the active-site strands within the multiple sequence alignment.

Analyzing the motions of each identified loop region within the MD trajectory narrowed the search down to three primary loops for each structure. These corresponded with regions of strong fluctuations in the RMSF analysis and are highlighted in green in the RMSF plot for P-As and T-Pf (Figure 4b,d, respectively). The loops and active sites for each chitinase structure are summarized in the RMSF plots (Figure S3), whereas the specific loop residue numbers and sequences corresponding to the X-ray structures are summarized in Table S2. For completeness, the RMSD plots of all loop regions, as identified from the RMSF data (all peaks in the 400 K plots), are presented in Figure S4.

From the proximity of these loop sequences to the active site, it is evident that the active sites of the psychrophilic chitinases (P-As and P-Mm) were flanked by these two adjacent loops, while the mesophilic M-Sp and M-Sm possessed floppy surface loops directly before and after the active site, respectively (Figure S3 and Table S2). In the chitinases investigated, the loops were located away from the active site only in T-Pf. The thermophilic T-Pf active site was stably embedded within the structure and flanked by helices. There was only one surface loop exhibiting a large primary motion vector (residues 670–680), which never occluded the central cavity or came closer than ≈2.4 nm to the active site. Other than this loop, there were no other clearly identifiable floppy loop regions.

Additionally, from the visual analysis of the frames extracted at 100 ns intervals of the high-temperature simulated trajectories (Figure S5), it was clear that the central TIM barrel cavity is not distorted. This explains the RMSD stability of the active site, as shown in Figure S2, where a comparison of RMSD of the loops vs. active site data alone is not enough to appreciate this correlation. From this evidence alone, it is difficult to classify each structure in terms of thermal adaptation, as the experimental melting temperatures (T_m_) of these were within 52–55 °C for the psychro- and mesophilic chitinases, but the optimum activity temperature (T_opt_) varied over a much wider range (28 to 65 °C), implying that other structural parameters must be critical for thermoactivity.

**Figure 7:**
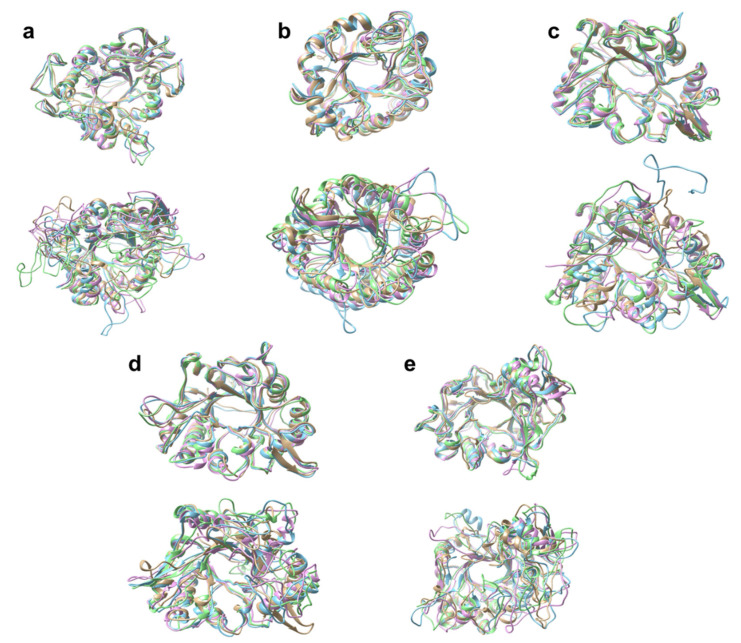
Superimposition of the frames extracted from the 300 K (upper row) and 400 K (lower row) trajectories at 0 ns (brown), 100 ns (blue), 200 ns (purple), and 300 ns (green): (**a**) P-As, (**b**) T-Pf, (**c**) M-Sm, (**d**) P-Mm, and (**e**) M-Sp. Occlusion of the central cavity by adjacent loops was evident in the 400 K structures of P-As and P-Mm, and to a lesser extent in M-Sm and M-Sp, whereas T-Pf retained its structure. The RMS deviations at 400 K between 0 ns and 300 ns were 1.259, 1.021, 1.266, 1.207, and 1.133 Å, respectively.

Visual analysis of trajectory frames at 100 ns intervals (Figure 7) showed the extent of the flexibility that the loop regions possess. Figure 7 shows the aligned and superimposed structures at both 300 K (upper) and 400 K (lower). From these frames, it can be clearly seen how the central cavity and helical regions remained largely intact, with the conformational changes localized mostly to the floppy loop regions, which is particularly evident for the cold-adapted chitinases. Considering that the number and location of floppy loop regions is critical for the stability, it may be concluded that obstruction of the central cavity by adjacent floppy loop regions (more in P-As and P-Mm, and to a lesser extent in M-Sm and M-Sp), with subsequent reduced access of the substrate to the active site, is a likely reason for reduced activity at higher temperatures.

### 2.5. Stabilizing and Destabilizing Hot-Spots in Chitinases

In order to predict which mutations can stabilize the psychrophilic and mesophilic chitinases, we subjected all chitinase structures to HoTMuSiC analysis (Table 2, light shaded). Additionally, we also analyzed all chitinases for destabilizing mutations that increase the flexibility with a view to enhancing the activity (Table 2, dark shaded). The top three mutations were identified based on all the loops and overall structure, excluding three catalytic site acidic residues. Our data show that the most stabilizing substitutions generally involved mutating a charged or polar residue into a more non-polar residue with a ΔT_m_ ranging from 1.9–3.9 K. Substituting glutamic acid for more hydrophobic residues was the most favored substitution, especially in the P-As (E357) and M-Sp (E372) pair. An exception was the V130E substitution in M-Sm.

In contrast with stabilizing mutations, the most destabilizing substitutions generally involved non-polar and aromatic residues being substituted by proline and, to a lesser extent, glycine residues, with the ΔT_m_ range (−6.7 to −13.1 K) being significantly more pronounced than for stabilizing mutations (1.9 to 3.9 K). The top potential target amino acids for each chitinase for both stabilization (charged/polar residues) and destabilization (non-polar and aromatic residues) followed this general trend. The sum of the ΔT_m_’s for potential target amino acids regarding destabilization (−78 to −146 K) was significantly more pronounced than for stabilizing mutations (11 to 31 K).

As for mobile loops, hot-spot residues, and substitutions, they generally followed a similar trend as for the overall structure (charged, polar, and less hydrophobic into more hydrophobic residues), whereas destabilization favored the reverse trend (Table 2). For 2DSK, the potential stabilizing mutations possessed the lowest stability increase, both overall and per residue, suggesting that the structure was already quite well adapted for stability. 4Q22 stood out for the potential to benefit most from loop-specific mutations, with 4AXN standing to lose the most from destabilization through the identified loops.

A mutagenesis study carried out on the residues of a surface mobile loop of chitinase B from *S. marcescens* (only 16% identity with M-SmC, 4AXN) showed that A234P resulted in a 5-fold increase in thermostability and a double mutant (G188A/A234P) gave a 10-fold increase, with concomitant increases in activity, whereas substitutions in other parts were neutral [26].

## 3. Discussion

Temperature is one of the most vital factors that influence life on Earth. Global temperatures can vary from −89 (Antarctica) to 400 °C (deep-sea hydrothermal vents), with metabolically active organisms found from −25 to 120 °C [27,28]. Thermally adapted extremophilic (psychrophilic and thermophilic) enzymes, including chitinases, have biotechnological applications in a wide range of industries. Psychrophilic (cold-adapted) enzymes have high intrinsic activity due to their higher structural flexibility with concomitant low stability, whereas thermophilic homologs have high stability due to their more rigid structure, which is accompanied by low intrinsic activity, in accordance with the activity–stability tradeoff [13,29]. Mesophilic homologs lie somewhere in between these two extremes. Cold-adapted enzymes are employed at low temperatures (4–25 °C), where they offer energy savings and avoid undesirable modifications of heat-sensitive reactants and products. Thermophilic homologs are instead employed at high temperatures for increased productivity, shorter process times, increased substrate solubility, lower medium viscosity, and a lower chance of contamination [19,27].

Although protein engineering has been undertaken for the enhancement of chitinase activity [30], only limited research has been targeted toward increasing their stability [23,26,31,32]. Additionally, the activity–stability trade-off limits the application of enzymes, such as chitinases.

Genetic and chemical modifications are beginning to show that the activity–stability trade-off can be avoided by modifying enzymes to have high stability without sacrificing activity [27,29]. However, a prerequisite for improving the stability of chitinases via rational design is the availability of sequence–structure–stability relationship information.

Thermophilic proteins are characterized by a higher number of non-covalent (salt bridges, H-bonds, aromatic–aromatic, cation-aromatic, hydrophobic–hydrophobic) and covalent (disulfide bridges) interactions, a compact hydrophobic core, lower surface hydrophobicity, amino acid bias toward proline and branched residues, oligomerization, and shorter and fewer surface loops [33]. On the other hand, low-temperature-adapted proteins show opposite trends, with concomitant flexible sites [13]. Unfolding has been shown to be initiated from these mobile loops, which once they have been identified computationally, can provide convenient targets for enhanced thermostabilization via site-directed mutagenesis [27,33,34].

By analyzing the sequence–structure–stability relationship and MD simulations in five thermally adapted GH-18 chitinase X-ray structures, we have identified structural regions that may confer stability in mesophilic and hyperthermophilic homologs and weak floppy sites in psychrophilic homologs. Furthermore, MD analysis was used to identify the most mobile surface loop from where unfolding was most likely to initiate. Additionally, we have suggested point mutations that can enhance the thermostability of less stable chitinase homologs.

Our data show that the key compositional elements that can confer lower flexibility with a concomitant higher stability in a mesophilic homolog included a lower acid/basic ratio, a lower Lys/Arg ratio, fewer Gly in α-helices, and a more basically charged C-cap, whereas trends in other compositional parameters were either the opposite or were similar (Figure 1, Table 1). For example, contrary to the general trend, mesophilic chitinases contained fewer Pro and more Gly residues in loops (Figure 1b) and larger cavities (Table 1), i.e., features that made the protein less stable [4,13]. Consistent with this, in a protein engineering study where ChiB-As Gly253 at the start of a mobile surface region was changed to Pro, the T_m_ of the modified protein was decreased [23].

The key structural elements that may confer higher stability to its mesophilic homolog included a greater accessible solvent area, greater buried and core hydrophobicity, and fewer disordered regions near the C-terminal, whereas trends in other structural features were the opposite. It is noteworthy that, contrary to general trends [13], the accessible solvent area was higher in mesophilic than its cold-adapted homologs (Figure 2a). It is interesting to note that the core hydrophobicity and solvent-accessible area in the P-Mm/M-Sm pair showed opposite trends as compared to the P-As/ChiSp pair.

The magnitude and nature (interactions between non-polar residues in the core and with the solvent) of the hydrophobic effect is the key to protein thermostability. More stable homologs have larger and branched non-polar residues, such as Ile, which can pack more efficiently (creates fewer cavities) inside the core with weaker and very short-range van der Waals interactions. Hydrophobic interactions are strongest around 20 °C and decrease at lower and higher temperatures, implying that these interactions can enhance stability at low-to-moderate temperatures. A smaller hydrophobic surface will stabilize a protein structure due to the lower tendency of water molecules to form low-entropy cage-like structures around nonpolar residues. Thus, thermostabilization involves a well-adjusted arrangement between the exposed and buried non-polar fraction, leading to decreased exposure to water and an optimal core packing [13,35]. The data presented in Figure 2 and Figure 3 and Table 1 regarding the hydrophobic fraction exposed to solvent, core/buried hydrophobicity, and hydrophobic interactions showed no clear trends that were consistent with the earlier reports, as discussed above [13,35]. For example, contrary to general trends [13], the buried hydrophobic area was higher in cold-adapted than in its mesophilic homolog (Figure 2, Table 1).

It is interesting to note that similar to unfolding at high temperatures, proteins are also denatured at low temperatures (mostly below the freezing point of water). Due to the lack of sufficient experimental evidence, the phenomenon of cold denaturation is not yet fully understood. The key mechanisms put forward to explain cold denaturation involve the interaction of water molecules with the buried hydrophobic and/or surface polar residues [13]. In the currently held view, water molecules coming in contact with the hydrophobic residues in the protein core results in the weakening of hydrophobic interactions at low temperatures, with concomitant enhanced fluctuations in the atoms’ movement and a decrease in the core compactness, leading to unfolding [36,37]. The proteins that are more thermostable were also found to be more resistant to cold denaturation, such as T-Pf, whereas the most cold-adapted (P-Mm) chitinase was expected to be the most susceptible to cold-denaturation (Table 1).

Higher numbers of interactions have been correlated with higher stability [13,27,29,33]. Mesophilic chitinases show higher numbers of hydrophobic interactions, whereas all other interactions were lower than their psychrophilic homologs (Figure 3). However, in the case of salt bridges and π–sulfur interactions, only M-Sm showed more interactions than P-Mm. (Figure 3, Table S1). Compared to mesophilic chitinases, both psychrophilic homologs showed a slightly higher number of total H-bonds per 100 residues, as determined using the PIC tool [38] (Figure 3a,b, Table S1). Similar results were obtained when the optimal H-bonding network was determined using the WHATIF tool [39]. The optimal H-bonding network is an extensive arrangement of one-to-one and bifurcated H-bonds that interconnect residues via other residues and water molecules [39]. A higher number of hydrogen bonds has been correlated with higher protein stability [40]. It is estimated that for a 10 °C rise in thermostability, an average of ≈13 H-bonds and salt bridges per chain are added. In a few protein families, H-bonds did not increase with enhanced thermostability; however, in these cases, an increase in salt bridges compensated for reduced H-bonds [41]. In contrast to the published results, this work implies that in thermally adapted chitinases (P-Mm vs. M-Sm and P-As vs. M-Sp) and T-Pf, H-bonds were not responsible for the higher stability of mesophilic and thermophilic chitinases, whereas more salt bridges may confer thermostability only in the case of the M-Sm vs. P-Mm pair (Figure 3, Table S1). Second, the dimeric structure of the mesophilic chitinase with 88 inter-subunit interactions (Table S1) may lead to stabilization, as described for other thermostable proteins by Vielle and Zeikus [33].

The presence of more and longer loops has been implicated in the thermolability of proteins [4,13,33]. Mesophilic chitinases show slightly less loop content. Loops, amino, and carboxyl termini are commonly the regions with the highest thermal oscillations in a protein crystal structure and are likely to unfold the earliest during thermal unfolding [33]. The number and location (proximity to the active site) of floppy loops, as identified via the MD analysis, are correlated with the thermoactivity, as these loops may be necessary for unhindered substrate access to the active site and/or are involved in substrate binding at lower temperatures.

However, our results suggest that the presence of these large flexible loops may have become a liability at higher temperatures, where they tended to occlude the central cavity, subsequently reducing access to the active site. It is also important to note the distinctly different clustering of M-Sm (Figure 5, red) compared with the other chitinases, where the results for M-Sm show two distinct distributions at ≈0.4 and ≈0.9 nm. As mentioned above, while the naturally dimeric M-Sm had greater stability than its cold-adapted monomeric homolog P-Mm, these simulations were only performed with the monomer unit.

The thermophilic chitinase (T-Pf) showed only a 12–18% sequence identity with the four other chitinases; therefore, exact comparisons between T-Pf and other homologs were not rigorous. However, our data show that the extreme stability of T-Pf was not due to any compositional feature (Figure 1 and Figure 2, Table 1) or more electrostatic interactions (Figure 3, Table S1). With respect to the structural features, a higher content of α-helical structures, lower content of random coil structures, higher core hydrophobicity, buried non-polar residues, and fewer core glycine residues were indicated as possible reasons for its higher stability. This was supported by the MD simulation measurements, where the radius of gyration and SASA were the lowest and most stable compared to less stable chitinases.

Another structural feature that may have contributed to the stability of T-Pf was its dimeric structure with 29 inter-subunit interactions. It is noteworthy that dimeric M-Sm had 88 inter-subunit interactions without being as stable as T-Pf (Table S1). Although the total loops and proportion of residues found in loops among T-Pf and other homologs were not very different, T-Pf contained only 3 loops longer than 10 residues. In T-Pf, a critical salt-bridge (D116-R175) existed between the active-site strand and an adjacent strand that was not present in the other chitinases. Stability analysis upon mutagenesis using the HoTMuSiC computational tool suggested that substitution of Arg581 with Pro, Ala, Gly, or Lys could destabilize the protein by −6, −4, −3.7, and −2.5 K, respectively. This is indicative of the importance of this salt bridge in the stability of T-Pf.

## 4. Materials and Methods

### 4.1. Sequence and Structural Analysis

The coordinates of the X-ray structures and protein sequences of the cold-adapted *M. marina* (P-Mm; PDB, 4HMC) [10] and its mesophilic homolog from *S. marcescens* (M-Sm; PDB, 4AXN) [42], cold-adapted *Arthrobacter* Tad20 (ChiB-As; PDB, 1KFW) and its mesophilic homolog from *S. proteamaculans* (M-Sp; PDB, 4Q22), and hyperthermophilic chitinase from *P. furiosus* (T-Pf; PDB, 2DSK) [12] belonging to GH-18 family were obtained from the PDB (Protein Data Bank) and NCBI (National Center for Biotechnology Information) data banks. All chitinases were composed of catalytic domains, except P-Mm, which consisted of catalytic, linker, and chitin-binding domains. Therefore, the P-Mm structure was truncated at the C-terminal end to remove the linker and binding domains (catalytic domain, G23–H339) for meaningful comparisons.

The computational analyses of chitinases were based on their amino acid compositions, sequences, and X-ray structures. The sequence-based analysis consisted of physico-chemical properties and amino acid compositional analysis based on the ProtParam tool (http://web.expasy.org/protparam/) and PDB DeepView. The disordered regions in chitinases were estimated by using metaPrDOS (http://prdos.hgc.jp/cgi-bin/meta/top.cgi [43]). The various bioinformatics tools used for the analysis of X-ray structures of chitinases are as follows. The pairwise multiple alignments of chitinases to identify conserved residues was performed with Ω-CLUSTAL (http://www.ebi.ac.uk/Tools/msa/clustalo/ [44]). Total intra- and inter-subunit interactions (disulfide bridges, H-bonds, salt bridges, hydrophobic interactions, cation–π and π–π interactions, etc) and solvent accessibility were estimated using WHATIF (http://swift.cmbi.ru.nl/servers/html/index.html [45]), PIC (Protein Interaction Calculator, http://pic.mbu.iisc.ernet.in/job.html, [38]), and CaPTURE (cation–π interactions in protein structures, http://capture.caltech.edu/ [46]). All these tools were based on the work by Burley and Petsko (π–π interactions), Overington et al. and Hooft et al. (optimal H-bonding), Reid et al. (cation–sulfur interactions), Kyte J. and Doolittle (hydrophobic interactions), and Hubbard and Thornton (solvent accessibility) [39,47,48,49,50,51]. The pockets and cavities in the chitinase structures were estimated using fpocket (http://mobyle.rpbs.univ-paris-diderot.fr/cgi-bin/portal.py?form=mdpocket#forms::fpocket [52]) and CASTp (http://sts.bioengr.uic.edu/castp/calculation.php [53]) tools, respectively. Prediction of protein stability after mutations were analyzed using the HoTMuSiC tool available at https://soft.dezyme.com/ [54,55].

### 4.2. Molecular Dynamics Simulation and Analysis

MD simulations were performed using GROMACS 2020.3 [56] with the CHARMM36 (jul2020) force field [57] and solvated with the TIP3P water model [58]. The protein was solvated with 100 mM NaCl to neutralize the charge in a dodecahedral box with a minimum distance of 1.0 nm to the box’s edge. The initial coordinates of the structures were extracted from the PDB crystal structures using the following PDB codes: P-As (1KFW [59]), T-Pf (2DSK [12]), M-Sm (4AXN [42]), P-Mm (4HMC [10]), and M-Sp (4Q22 [60]). MD simulations were performed at two temperatures (300 and 400 K) for a total of 300 nanoseconds each, with a time step of 2 fs and coordinates recorded every 10 ps for a total of 30,000 frames. All MD simulations and processing were run on personal hardware owned and operated by the first author (D.L.A.). Data analysis was performed with the standard GROMACS utilities to process the trajectories, Xmgrace and KDE LabPlot were used to generate the plots, and the solvent-accessible surface area was calculated using the SASA algorithm [61,62]. Visual Molecular Dynamics 1.9.4 [63] was used for the visualization of the trajectories and generating images. Clustering analysis was performed with the gromos algorithm using a 0.5 nm cutoff and a 5 ns stride [64].

## 5. Conclusions and Future Perspectives

More and longer surface loops have been implicated in decreasing the stability of proteins, including chitinases. Unfolding is suggested to be initiated from the mobile surface loops [4,33]; therefore, stabilizing these “weak spots” can lead to thermostabilization in chitinases [26]. An MD analysis of a mesophilic chitinase (T_opt_ = 50 °C) from *Thermomyces lanuginosus* revealed that even at higher temperatures, the stability of the protein was uniformly distributed throughout the structure [65]. It is important to note that the study was limited to a very short 10 ns simulation length. From our MD results, it can take anywhere from 20 to 100 ns for the RMSD to stabilize. In reality, these timescales are still too short to capture protein unfolding events, which are typically on the timescale of microseconds to milliseconds, a challenging timescale for contemporary MD simulations [66]. Instead, we chose to study the motion and interactions of the mobile surface loops with relation to the active site. What may be understood from the MD simulations is that the prevalence and flexibility of loops adjacent to the active site is of importance in low-temperature adapted chitinases. Correlations with temperature dependence involves proximity and flexibility of the loops to the active site. We hypothesize that the presence of large flexible loops occluded the active site at higher temperatures, leading to reduced catalytic activity.

Our data predicted substitutions (Table 2) that were consistent with the site-directed mutagenesis study indicating that A→P substitutions can be stabilizing due to the rigidity that proline imparts to the loop [26]. This is achieved by only those proline substitutions that decrease the conformational entropy of the unfolded state without imparting strain, steric clash, or breaking electrostatic interactions [33]. In contrast, proline residues within the α-helix are destabilizing due to the disruption of the secondary structure [67]. This insight will allow more directed studies to improve the stability–activity properties of this important class of enzymes by stabilizing mobile loops via protein engineering.

**Figure 8:**
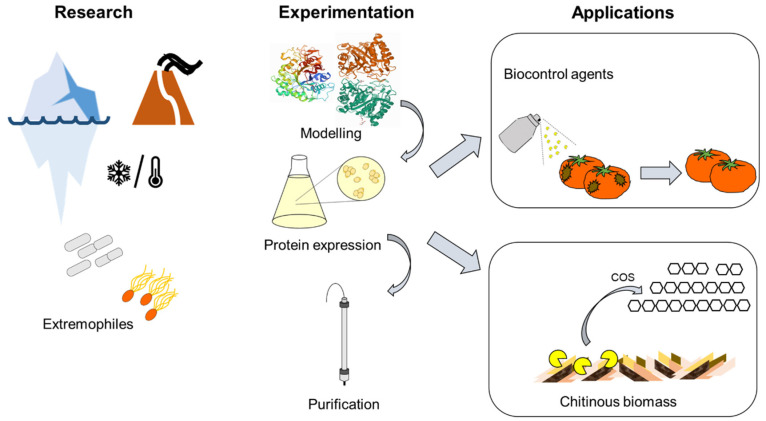
Perspectives linked to the agricultural uses of improved chitinases. Research involving the modeling of extremozymes and rational design can lead to superior catalysts in terms of stability. Such enzymes can be expressed in heterologous hosts, purified, and used as biocontrol agents or to degrade chitinous waste to yield chitooligosaccharides (COSs). COSs can in turn be applied as elicitors to improve biotic stress responses in crops.

The results obtained herein with the aid of computational and MD simulations will inspire future research that is aimed at improving the stability and thermoactivity of chitinases and enhance their uses in different industrial applications. We believe it is important to emphasize one specific example of application, i.e., the agricultural use as biocontrol agents against biotic stresses in plants (Figure 8). Indeed, a study successfully used a plant-purified chitinase (GH19) as a biocontrol agent to alleviate the stress symptoms on powdery-mildew-infected strawberry [68]. Chitinases produced by chitinolytic organisms [69,70] are biodegradable and present interesting alternatives for fungicide uses.

One major drawback preventing a full exploitation of chitinases as biocontrol agents is their slow action mode, high instability, and reduced persistence in the environment [70]. By coupling in silico studies with rational design and chemical modification, improved chitinases can be obtained that show higher stability without a loss of activity for use as crop-protecting formulations. The industrial-scale production of such improved enzymes could potentially be achieved using robust commercial expression systems, such as *Pichia pastoris*, which was shown to be a suitable eukaryotic host for the production of enzymes, including extremozymes [71]. The encapsulation of the chitinases in nanoparticles (NPs) could provide an additional way to increase their stability. In this respect, the recent use of lignin NPs loaded with fungicide has proved to be an efficient approach to treating the grapevine trunk disease Esca [72]; these NPs can be injected directly into the trunk and their degradation with the subsequent release of fungicides will be triggered only upon infection following the secretion of fungal ligninolytic enzymes. Chitinases loaded in BSA (bovine serum albumin) NPs have been proven to be effective against *Alternaria alternata* [73]. Improved chitinases loaded in NPs could also potentially be used for similar agricultural biocontrol applications.

An additional industrial application is the production of COSs from chitinous waste, such as fungal mycelia and crustacean shells. COSs are used in the nutraceutical sector due to their ability to lower cholesterol and blood pressure [74,75], as well as in agriculture as biostimulants, e.g., any substance or microorganism that improves crop quality, plant stress resistance, and nutrients’ uptake [76]. Chitin and its derivatives stimulate plant growth [77], protect against (a)biotic stress [78,79,80], and have been used as coating agents with biostimulatory effects [81].

Chitin derivatives are also used in the preparation of contact lenses, artificial skin, drug-delivery materials, and surgical stitches [6]. For example, food-grade COS has been prepared using a recombinant chitinase from *Lactococcus lactis* expressed in *Escherichia coli* BL21 (DE3) [76]. Improved chitinases obtained via computer-assisted rational design (e.g., engineered to produce COS with degrees of polymerization >6 that are the most effective as plant elicitors, i.e., molecules triggering defense responses) that are able to withstand higher temperatures or high pH may be used directly after biomass pre-treatment (step during which the inter- and intramolecular H-bonds in chitin are disrupted). COSs obtained from chitinous biomass hydrolysis can be applied to improve the innate immunity in plants via a mechanism that allows for binding to membrane receptors [82], and hence makes them more resistant to biotic stresses.

## Figures and Tables

**Table 1:** Properties and compositional/structural features of thermally adapted chitinases.

Chitinases Features	P-Mm	M-Sm	P-As	M-Sp	T-Pf
*Stability:	[8]	[22]	[7,23]	[24]	[25]
T_opt_ (°C)	28 (pH 5)	65 (pH 7)	30	40–45 (pH 6)	95 (pH 6)
T_m_ (°C)	55 (pH 8)	55 (pH 7)	52 (pH 7.3)	52 (pH 6)	114 (pH 6)
Irreversible thermal	50% activity loss in 10	50% activity loss in 26	90% activity loss in 60	-	-
inactivation	min at 60 °C	min at 57 °C	min at 45 °C	-	-
pH_opt_	5.0	5.0	7.3	6.0	6.0
Subunits ↑	1	2	1	1	2
Total residues	317	328	435	398	311
Isoelectric point (pI) ↑	4.50	5.20	6.60	6.20	4.75
Pro (N-cap of an α-helix) ↑	0	1	1	2	1
Pro (middle of an α-helix) ↓	2	2	0	1	0
Charged N-cap:					
Total (acidic) ↑	7	9	3	4	9
Total (basic) ↓	1	4	2	7	1
Charged C-cap:					
Total (acidic) ↓	2	2	1	0	4
Total (basic) ↑	1	1	4	7	7
^1^ Total isoleucine residues	26	23	13	16	22
Residues inside the core ↑	7.0	5.0	5	4	4
^1^ Total glycine residues	28	27	55	32	23
Residues inside the core ↓	4	5	6	5	3
^2^ Cavities (volume, Å^3^) ↓	1001	2155	4206	4135	1495
^3^ Total pockets and cavities	5	14	14	16	11
^4^ Disordered regions:					
N-terminal residues	7	22	5	4	7
C-terminal residues	5	5	12	5	16
Within sequence	-	-	-	DAAKNPV	PTGEVSP

M, mesophilic; T, thermophilic. Mm, *M. marina*; Sm, *S. marcescens;* As, *Arthrobacter* sp.; Sp, *S. proteamaculans*; Pf, *P. furiosus.* All features were analyzed using the ProtParam tool or PDB DeepView. ↑, higher value of the parameter is associated with higher stability; ↓, higher value of the parameter is associated with lower stability. T_opt_, optimum temperature; T_m_, melting temperature. ^1^, using WHATIF; ^2^, using CASTp; ^3^, using fpocket; ^4^, using metaPrDOS, which included PrDOS, DISOPRED2, DisEMBL, DISPROT, DISpro, IUPred, POODLE-S, and DISOclust. *Stability, pH_opt_ data taken from the Enzyme Database (BRENDA).

**Table 2:** Prediction of the stabilizing and destabilizing mutations in chitinases.

Chitinases (Mutations)		P-Mm (4HMC)	M-Sm (4AXN)	P-As (1KFW)	M-Sp (4Q22)	T-Pf (2DSK)
Most stabilizing mutations	Overall structure	N67I (2.7)	A251P (2.8)	E357I (3.5)	E372I (3.9)	E482I (2.3)
		N67V (2.6)	V130E (2.5)	E357V (3.2)	E372L (3.4)	S556C (2.1)
		N216Y (2.5)	Q206P (2.4)	E357Y (3.2)	E372V (3.3)	E482V (1.9)
	Mobile Loop, L1	E123V (0.9)	A36P (1.6)	A103P (0.7)	E68C (2.5)	S556C (2.1)
		E123I (0.8)	G41W (1.1)	W106P (0.6)	E68I (2.1)	S556F (1.6)
		E123T (0.8)	G41F (0.9)	A94P (0.6)	E68V (2.0)	S556I (1.6)
	Mobile Loop, L2	K160G (0.8)	T152R (1.4)	N170P (0.5)	G41P (1.8)	T586Y (0.6)
		K160C (0.3)	A148G (1.3)	E172G (0.4)	G32F (1.4)	T586F (0.6)
		K160P (0.3)	T152E (1.2)	E172N (0.4)	G32W (1.3)	T586W (0.4)
	Mobile Loop, L3	N323G (0.7)	A268W (0.3)	N203M (1.0)	S165I (1.3)	G672C (0.5)
		A322D (0.7)	A270L (0.2)	N203L (0.9)	E164W (1.2)	R682Y (0.4)
		A322T (0.6)	A268F (0.1)	N203V (0.9)	V156P (1.2)	G672V (0.3)
Potential targets for enhancing stability (sum of all mutation scores for the residue)	Overall structure	N67 (15.0)	K68 (21)	E357 (31)	E372 (24)	G659 (12)
		N216 (12)	V130 (14)	D190 (19)	A18 (20)	S556 (9.8)
		G35 (11)	I17 (11)	G56 (19)	E68 (18)	E482 (8.4)
	Mobile Loop, L1	E123 (6.2)	A36 (4.4)	A103 (0.8)	E68 (18.4)	S556 (9.8)
		D128 (2.7)	G41 (3.1)	W106 (0.7)	E65 (7.5)	G564 (5.5)
		D130 (1.5)	A35 (1.5)	A94 (0.7)	N63 (2.5)	G565 (3.2)
	Mobile Loop, L2	K160 (1.8)	T152 (7.8)	E172 (1.8)	G32 (5.2)	T586 (1.6)
		A155 (0.3)	A143 (3.5)	N170 (0.7)	A37 (5.2)	D588 (0.3)
		Q154 (0.1)	A148 (2.2)	A179 (0.4)	T36 (3.3)	M587 (0.1)
	Mobile Loop, L3	A322 (3.0)	A268 (0.5)	N203 (4.9)	E164 (5.7)	G672 (0.9)
		N324 (2.6)	A270 (0.2)	G199 (1.0)	S165 (4.6)	L680 (0.6)
		N323 (0.7)	N266 (0.1)	G204 (0.9)	V156 (3.6)	T674 (0.6)
Most destabilizing mutations	Overall structure	L269P (−7.7)	G132P (−13.1)	L321P (−9.0)	Y276P (−11.4)	I485G (−10.4)
		Y220P (−6.9)	F258P (−12.8)	I187D (−8.9)	F343P (−9.5)	Y520P (−9.7)
		I215P (−6.7)	F202P (−12.3)	I187G (−8.6)	L177P (−9.4)	F523G (−9.7)
	Mobile Loop, L1	I122D (−5.4)	G41E (−5.2)	L110P (−6.1)	I61G (−7.4)	G561P (−7.3)
		I122P (−5.3)	G41K (−4.4)	Y93E (−5.9)	I61D (−7.2)	G561E (−7.1)
		I122G (−5.2)	Q44P (−3.9)	Y93P (−5.6)	I61P (−6.9)	G561D (−7.0)
	Mobile Loop, L2	A156P (−3.4)	A144P (−5.4)	G166P (−6.9)	G38P (−7.2)	Y589G (−6.3)
		A156K (−3.1)	K151G (−5.2)	G166E (−6.9)	G33P (−6.8)	Y589E (−5.2)
		I157G (−2.8)	N150P (−5.0)	G166D (−6.0)	L46P (−5.7)	Y589A (−4.9)
	Mobile Loop, L3	G318P (−5.5)	N265P (−7.9)	W193G (−5.0)	V163P (−6.9)	G683P (−7.8)
		F330P (−5.3)	A269P (−5.8)	P194G (−4.2)	G158P (−6.5)	G683E (−6.1)
		I331P (−5.3)	N265G (−5.7)	W193K (−4.1)	G158V (−6.3)	H681P (−5.7)
Potential targets that could lead to destabilization (sum of all mutation scores for the residue)	Overall structure	Y220 (−79)	G132 (−146)	I187 (−106)	F394 (−113)	F523 (−124)
		I215 (−79)	F202 (−137)	I51 (−102)	I148 (−111)	F418 (−115)
		V111 (−78)	G135 (−125)	W332 (−101)	W204 (−109)	F663 (−114)
	Mobile Loop, L1	I122 (−68)	G41 (−38)	Y93 (−68.3)	I61 (−93)	G561 (−108)
		L124 (−59)	G45 (−32)	L110 (−54.0)	Y62 (−73)	L562 (−62)
		G116 (−52)	Y42 (−26)	G101 (−42.4)	L73 (−73)	P558 (−50.4)
	Mobile Loop, L2	A156 (−40.3)	K151 (−43)	G166 (−89)	G38 (−80.6)	Y589 (−68.4)
		I157 (−29.2)	N150 (−40)	I183 (−59)	G33 (−62.5)	S594 (−41.0)
		A159 (−17.4)	A147 (−36)	L168 (−52)	I43 (−49.7)	T586 (−36.5)
	Mobile Loop, L3	F330 (−67.3)	N265 (−68)	W193 (−53.7)	G158 (−70)	G683 (−82.8)
		I331 (−56.6)	V274 (−65)	G202 (−37.5)	V163 (−63)	G675 (−54.5)
		G318 (−39.1)	Y273 (−52)	G195 (−35.4)	Y154 (−42)	R682 (−44.6)

Values found using the HoTMuSiC tool. Values that are given in parenthesis represent the mutation scores as ΔT_m_ (Kelvin, K). ΔT_m_ > 0 and ΔT_m_ < 0 means stabilizing and destabilizing mutations, respectively. NA, not applicable. Light shade, stabilizing; dark shade, destabilizing. Only data for the top three mutations are given.

## Data Availability

The data presented in this study are available in article and Supplementary Material.

## Supplementary Materials

The following are available online, Table S1: Various interactions in thermally adapted chitinases; Table S2: Summary of the active site and identified loop sequences; Figure S1: Secondary structure analysis; Figure S2: Root-mean-square deviation (RMSD), solvent-accessible surface area (SASA), and radius of gyration plots; Figure S3: Root-mean-square fluctuations (RMSF) plots with key loops and active sites; Figure S4: RMSD of all identified loops and active sites; Figure S5: Frames extracted from 400 K molecular dynamics (MD) simulations.

## Sample Availability

Not available.
