# Supplementary material for: Computational Analysis of Thermal Adaptation in Extremophilic Chitinases: The Achilles’ Heel in Protein Structure and Industrial Utilization
Source: Molecules. 2021 Jan 29;26(3):707. doi: 10.3390/molecules26030707 (PMC7866400; doi:10.3390/molecules26030707)
Supplement: Supplementary file 1 [file molecules-26-00707-s001.pdf]

## Electronic Supplementary Information:

# Computational Analysis of Thermally-Adapted Extremophilic Chitinases: the Achilles Heel in Protein Structure and Industrial Utilization

**Dale L. Ang [1,†], Mubasher Zahir Hoque [2,†], Md. Abir Hossain [2,3], Gea Guerriero [4,*], Roberto Berni [5], Jean-Francois Hausman [4], Saleem A Bokhari [6], Wallace J. Bridge [7] and Khawar Sohail Siddiqui [7,*]**

[1]Department of Molecular Sciences, Macquarie University, North Ryde, New South Wales, NSW 2109, Australia; dale.ang@mq.edu.au

[2]Bio-Bio-1 Research Foundation, Sangskriti Bikash Kendra Bhaban, 1/E/1 Poribagh, Dhaka 1000, Bangladesh; mubasher_zahir856@hotmail.com (M.Z.); mdabir14@gmail.com (M.A.H.)

[3]Department of Biochemistry and Microbiology, North South University, Plot 15, Block B, Bashundhara, Dhaka 1229, Bangladesh

[4]Research and Innovation Department, Luxembourg Institute of Science and Technology, 5, rue Bommel, Z.A.E. Robert Steichen, L-4940, Hautcharage, Luxembourg; jean-francois.hausman@list.lu

[5]TERRA Teaching and Research Center, Gembloux Agro-Bio Tech, University of Liège, 5030 Gembloux, Belgium; Roberto.Berni@uliege.be

[6]Biosciences Department, COMSATS University Islamabad, Park Road, Islamabad, 45550, Pakistan; saleem.a.bokhari@comsats.edu.pk

[7]School of Biotechnology and Biomolecular Sciences (BABS), University of New South Wales, Sydney, NSW, 2052, Australia; wj.bridge@unsw.edu.au; z3030204@ad.unsw.edu.au

*Correspondence: gea.guerriero@list.lu (G.G.); sohailsiddiqui1995@yahoo.com (K.S.S.); Tel: +352-2758885096 (G.G.); +61-2-9385-5193 (K.S.S.)

†These authors contributed equally to this work.

# Table of contents

**Fig. S1.** Secondary structure analysis 3

**Fig. S2.** RMSD, SASA, Radius of gyration plots 6

**Fig. S3.** RMSF plots with key loops and active site highlighted 7

**Fig. S4.** RMSD of all identified loops and active sites 7

**Fig. S5.** Frames extracted from 400 K MD simulations 9

**Table S1.** Various interactions in thermally adapted chitinases 9

**Table S2.** Active site and loop sequences 10

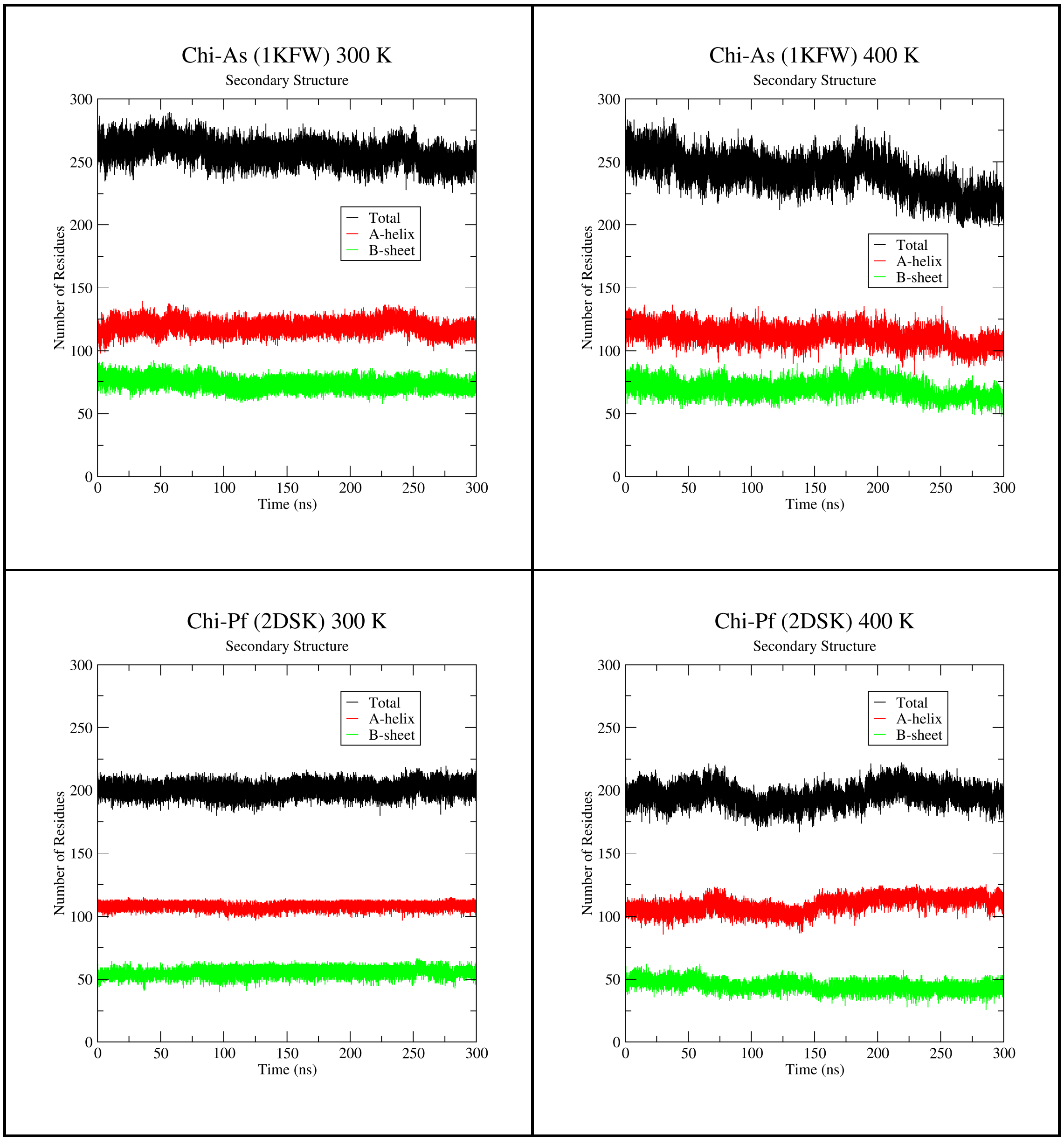
Chi-As (1KFW) 300 K
Secondary Structure
Chi-As (1KFW) 400 K
Secondary Structure
Chi-Pf (2DSK) 300 K
Secondary Structure
Chi-Pf (2DSK) 400 K
Secondary Structure
Number of Residues
Time (ns)
Total
A-helix
B-sheet
0
50
100
150
200
250
300

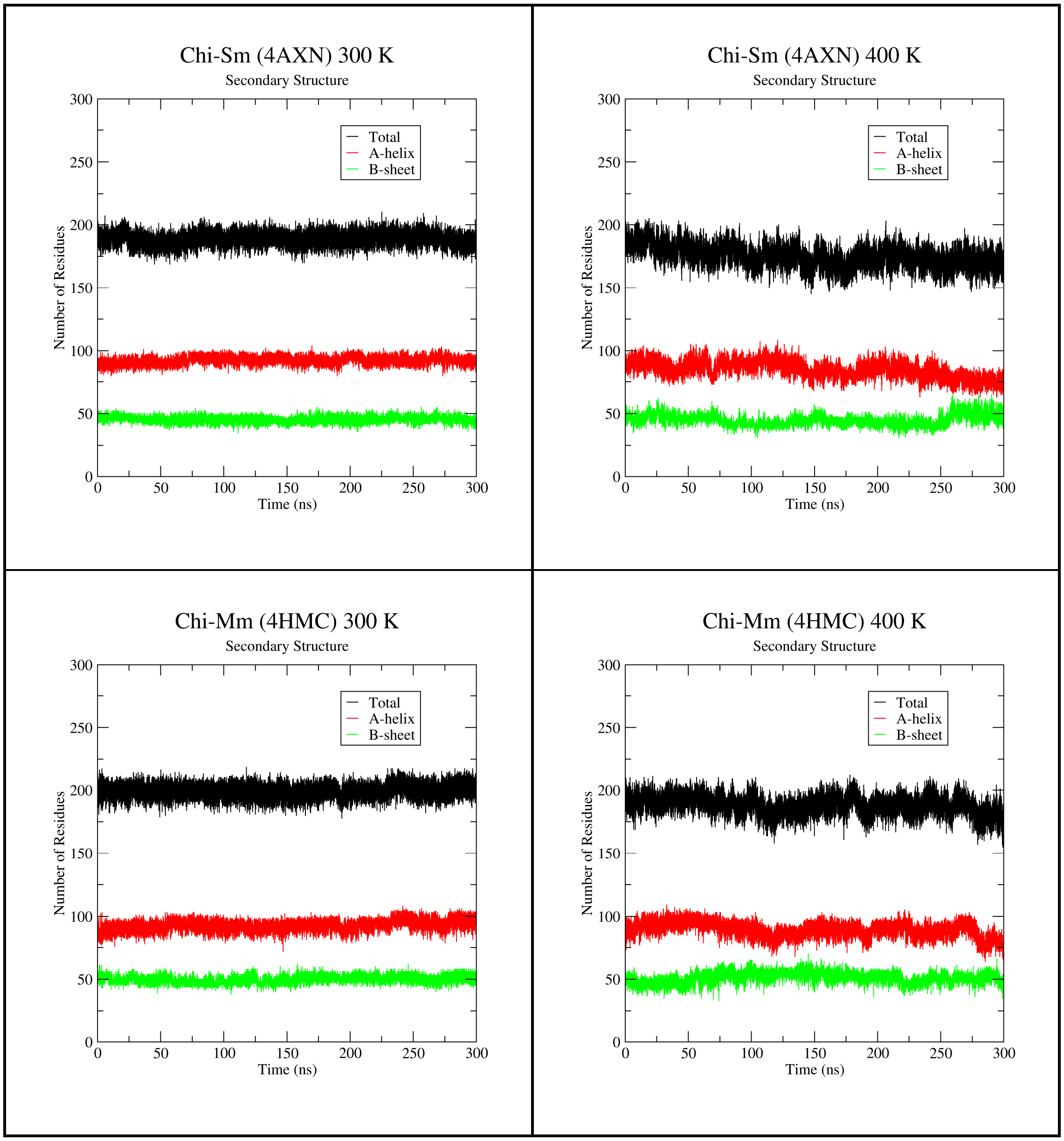
Chi-Sm (4AXN) 300 K
Secondary Structure
Chi-Sm (4AXN) 400 K
Secondary Structure
Chi-Mm (4HMC) 300 K
Secondary Structure
Chi-Mm (4HMC) 400 K
Secondary Structure
Total
A-helix
B-sheet
Number of Residues
Time (ns)
0
50
100
150
200
250
300

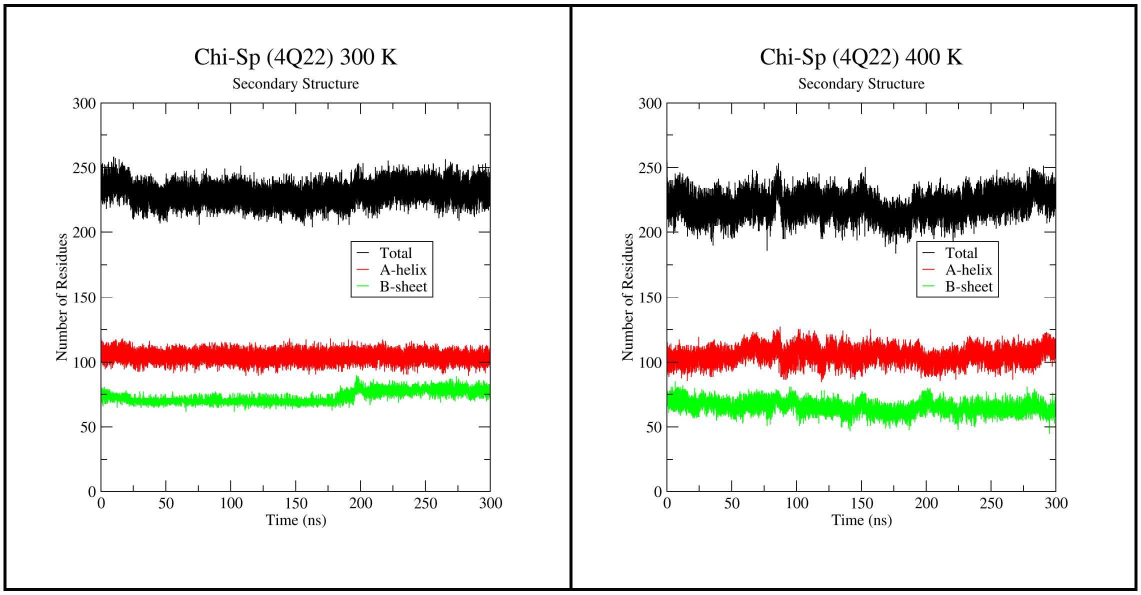


**Figure S1.** Secondary structure content for all proteins at 300 K and 400 K as a function of simulation time.

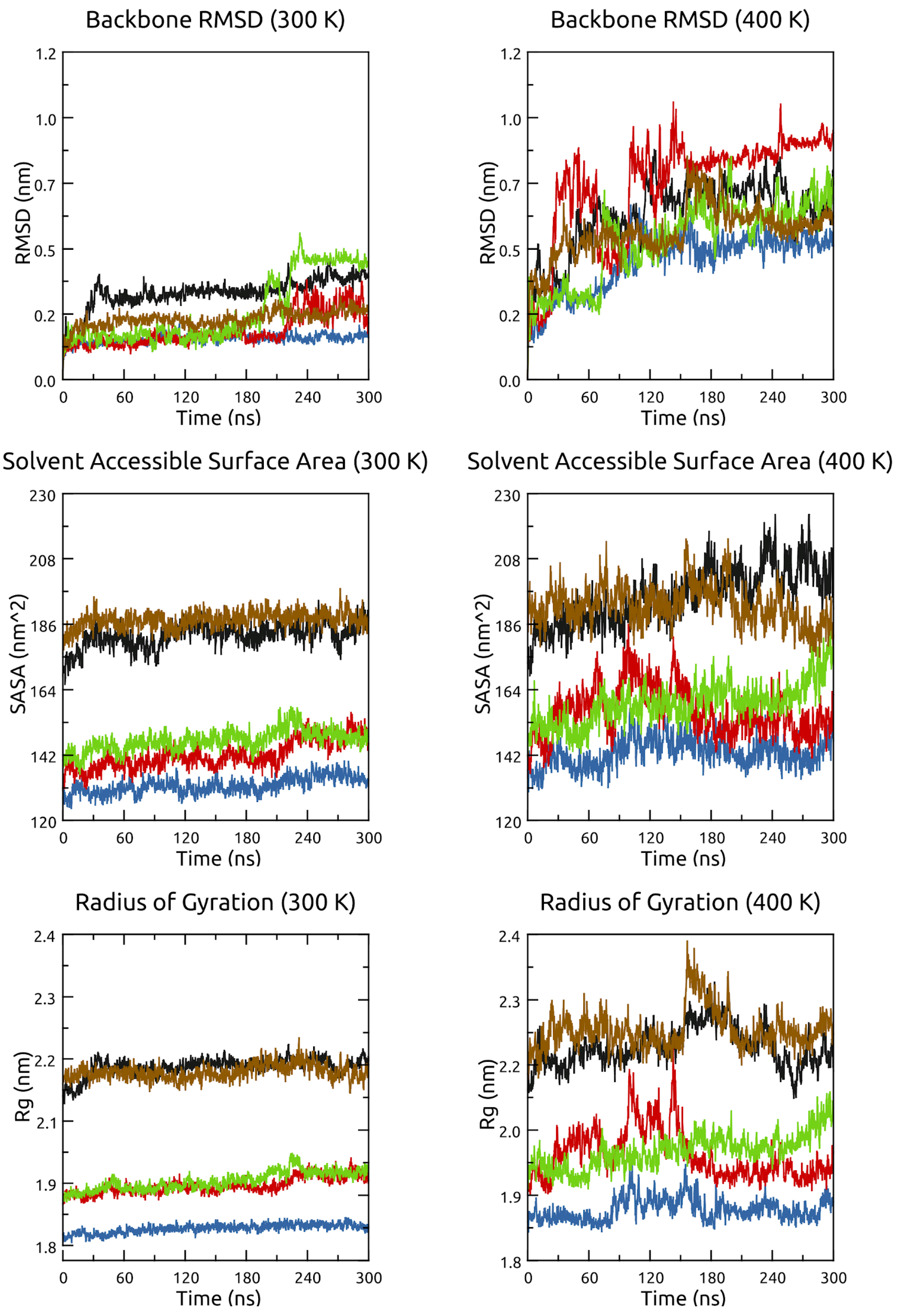


**Figure S2.** Root mean square deviation (RMSD), solvent accessible surface area (SASA), and radius of gyration (Rg) as a function of simulation time. P-As 1KFW (black), T-Pf 2DSK (blue), M-Sm 4AXN (red), P-Mm 4HMC (green), M-Sp 4Q22 (brown).

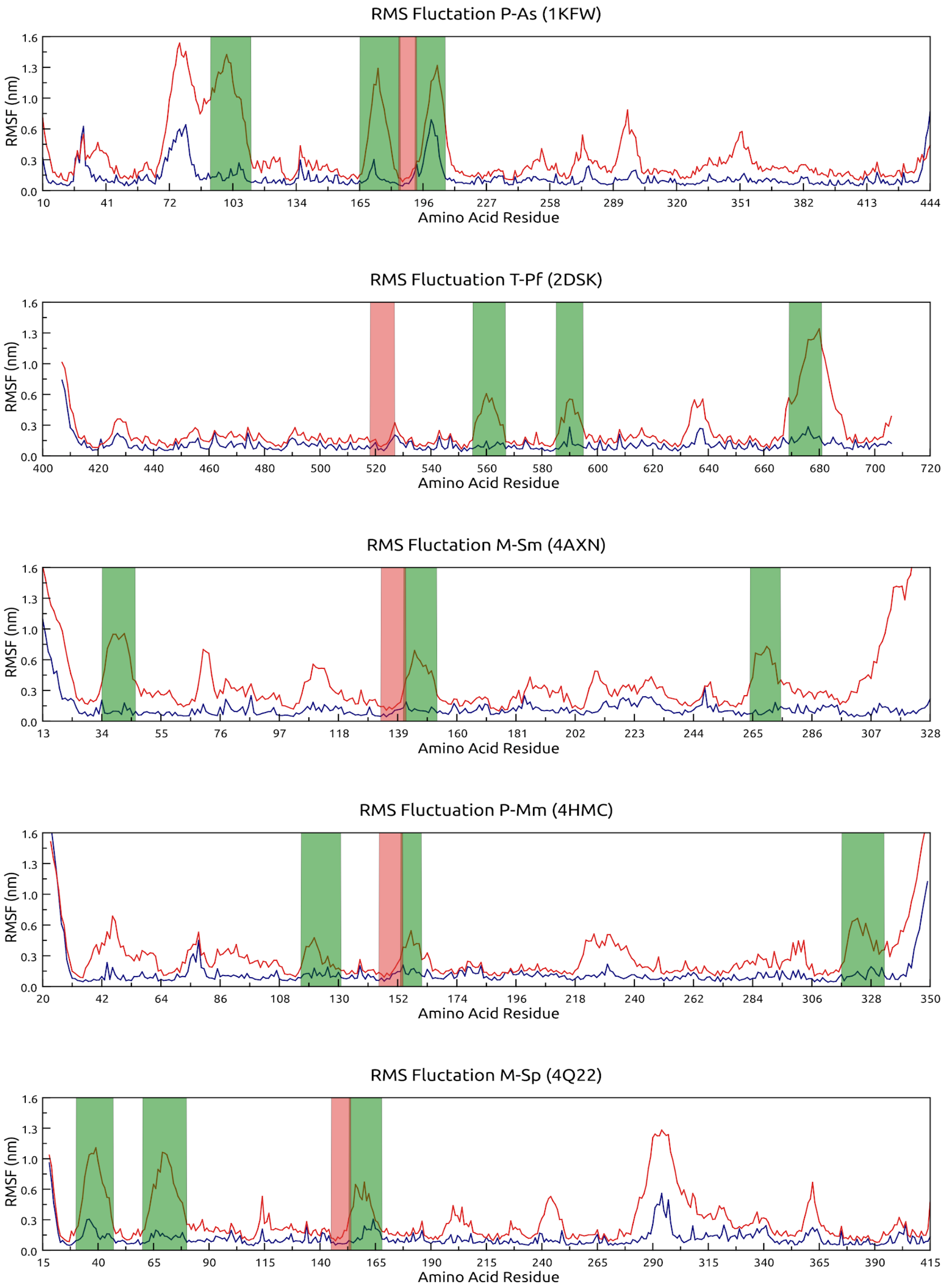


**Figure S3.** Root mean square fluctuations (RMSF) as a function of residue for 300K (blue) and 400 K (red). Floppy loop regions are highlighted in pale green, active site highlighted in pale red.

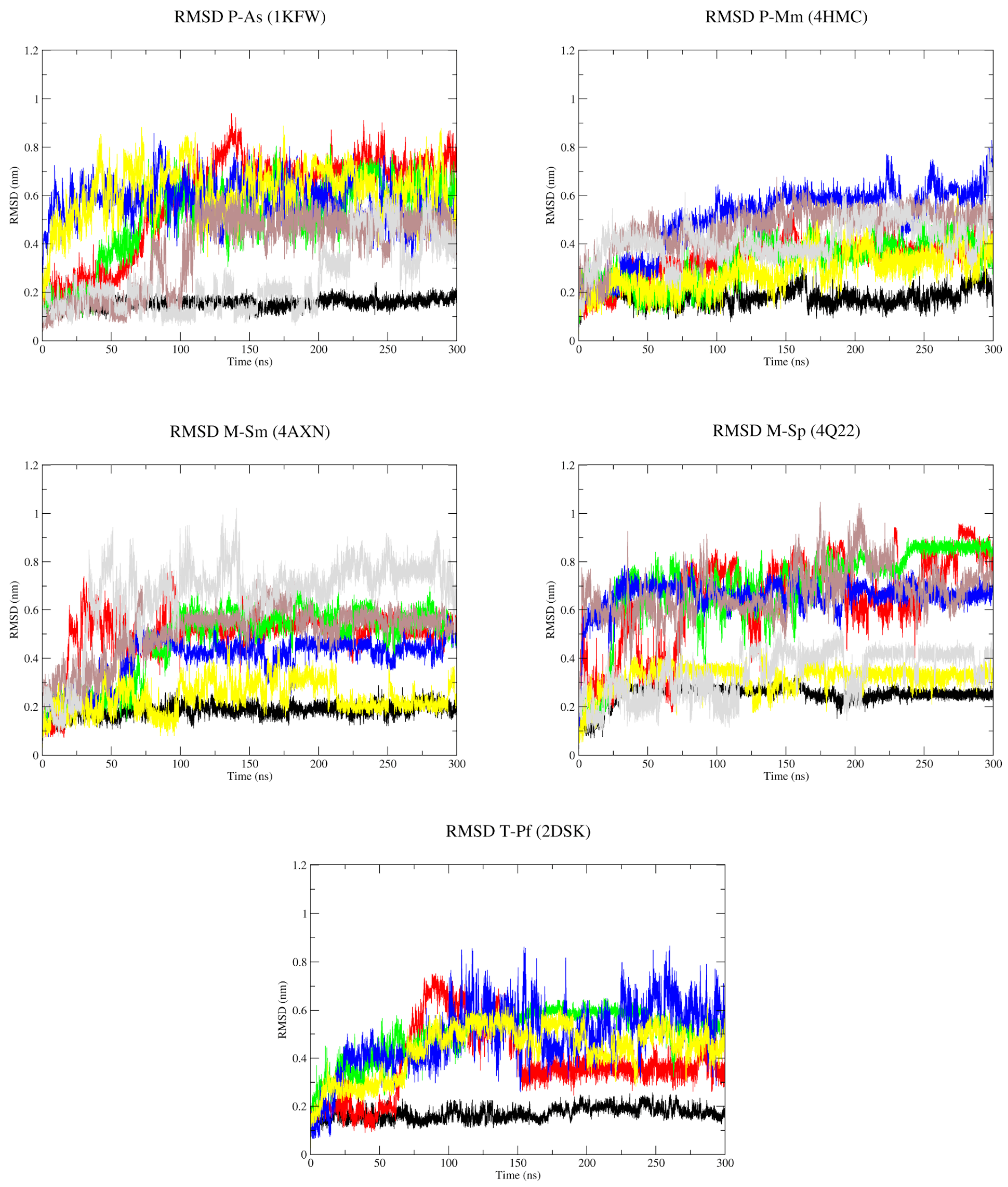


**Figure S4.** Root mean square deviation of loops (RMSD), and active site (black). Loop residue IDs: P-As: 93-111, 166-184, 193-206, 71-86, 293-300, 349-354; active site: 184-192. P-Mm: 117-130, 154-160, 318-332, 42-50, 73-92, 222-238; active site: 146-153. M-Sm: 35-45, 142-152, 265-274, 68-74, 106-114, 306-317; active site: 134-141. M-Sp: 31-46, 61-79, 154-167, 242-247, 283-309, 359-364; active site: 146-153. T-Pf: 556-566, 586-594, 670-680, 633-640; active site: 519-526. Colouring: red, green blue, yellow, brown, grey, respectively. Active site is in black.

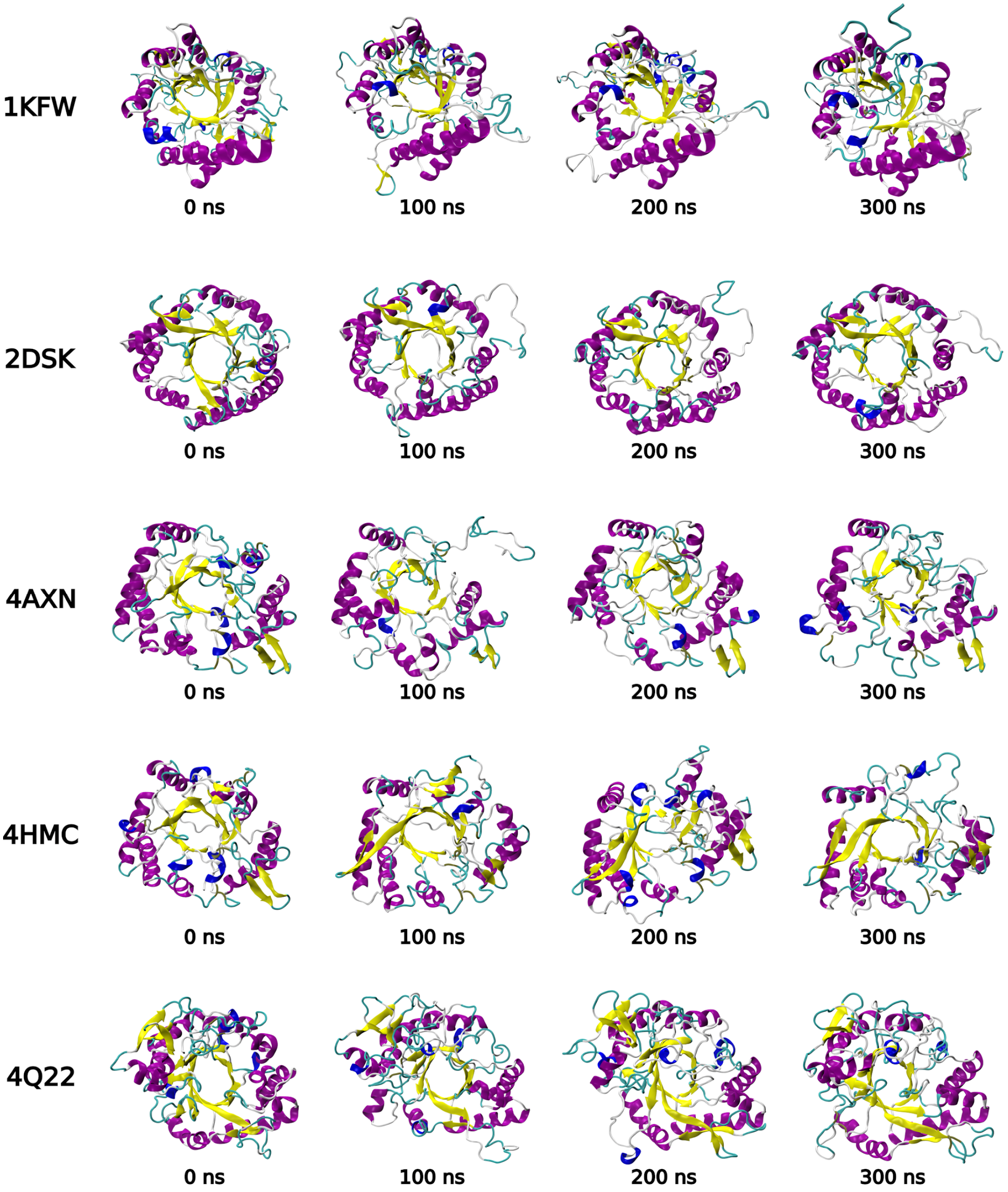


**Figure S5.** Conformations of proteins extracted at 0 ns, 100 ns, 200ns, and 300 ns from the trajectories of the 400 K simulations.

**Table S1.** Various interactions in thermally adapted chitinases.

| Chitinases<br>Features | **P-Mm** | **M-Sm** | **P-As** | **M-Sp** | **T-Pf** |
|---|---|---|---|---|---|
| [1]**H-bonds (dA:O, N = 3.5; S = 4.0 Å):** | | | | | |
| **Total:** | | | | | |
| Main chain-main chain | 387 | 377 | 497 | 478 | 392 |
| Main chain-side chain | 151 | 136 | 222 | 185 | 133 |
| Side chain-side chain | 132 | 115 | 194 | 123 | 107 |
| **Buried:** | | | | | |
| Main chain-main chain | 107 | 103 | 139 | 107 | 106 |
| Main chain-side chain | 36 | 40 | 41 | 38 | 35 |
| Side chain-side chain | 15 | 15 | 40 | 30 | 27 |
| **Exposed:** | | | | | |
| Main chain-main chain | 168 | 161 | 211 | 224 | 169 |
| Main chain-side chain | 81 | 59 | 107 | 91 | 68 |
| Side chain-side chain | 89 | 82 | 94 | 48 | 66 |
| [2]**Salt-bridges (within 4 Å):** | | | | | |
| **Total:** | | | | | |
| $COO^-$ →Lys | 4 | 7 | 12 | 5 | 5 |
| $COO^-$ →His | 0 | 0 | 2 | 1 | 1 |
| $COO^-$ →Arg | 5 | 8 | 7 | 8 | 7 |
| **Buried:** | | | | | |
| $COO^-$ →Lys | 0 | 0 | 1 | 1 | 0 |
| $COO^-$ →His | 0 | 0 | 0 | 0 | 0 |
| $COO^-$ →Arg | 0 | 0 | 0 | 0 | 2 |
| **Exposed:** | | | | | |
| $COO^-$ →Lys | 3 | 7 | 9 | 4 | 5 |
| $COO^-$ →His | 0 | 0 | 0 | 1 | 1 |
| $COO^-$ →Ar | 5 | 8 | 4 | 4 | 4 |
| [2]**Protein-protein interchain** | **NA** | **88** | **NA** | **NA** | **29** |
| **interactions** | **1** | 2 (A + B) | **1** | **1** | 2 (A + B) |
| Subunits ↑ | | 516 + 517 | | | 409 + 435 |
| Interface area (Å$^2$) | | 13 + 13 | | | 9 + 6 |
| Interface residues | | 77 | | | 22 |
| Non-bonded contacts | | 8 | | | 4 |
| H-bonds | | 0 | | | 1 |
| Cation-pi interactions | | 0 | | | 0 |
| Salt-bridges | | | | | |

M, mesophilic; T, thermophilic. Mm, *M. marina*; Sm, *S. marcescens;* As, *Arthrobacter sp.;* Sp, *S. proteamaculans*; Pf, *P. furiosus.* 1, 2 using PIC and 3 using PDBsum.

**Table S2.** Summary of active site and primary loop sequences.

| Structure | Active site | Loop 1 | Loop 2 | Loop 3 |
|---|---|---|---|---|
| P-As (1KFW) | D185-E192 | 93-YAADKSVSGKADTWDQPLA-111 | 166-GNLPNFEGRGGAGAAAGI-183 | 193-WPGTNSGLAGNGVD-206 |
| T-Pf (2DSK) | T519-E526 | 556-SDPGIGLAGGY-566 | 586-TMDYYWTPS-594 | 670-HPGPTGEVSPL-688 |
| M-Sm (4AXN) | D134-E141 | 35-AAGASDGYQQG-45 | 142-QAAIGAANNKT-152 | 265-NNDAAATGYV-274 |
| P-Mm (4HMC) | D146-E153 | 117-GADAHIELTRGDED-130 | 154-QAAITAK-160 | 318-GTDAANNSYNQQFIK-332 |
| M-Sp (4Q22) | D146-E153 | 31-GGGDVTAGPGGDINKL-46 | 61-IYNDEKQETNPALKDPSRL-79 | 154-YPVNGAWGLVESQP-167 |
